# Modulation of Neutrophil Activity by Soluble Complement Cleavage Products—An In-Depth Analysis

**DOI:** 10.3390/cells11203297

**Published:** 2022-10-20

**Authors:** Lisa Wohlgemuth, Alexander Elias Paul Stratmann, Frederik Münnich, Stefan Bernhard, Bertram Dietrich Thomaß, Finn Münnich, Adam Omar Khalaf Mohamed, Marco Mannes, Christoph Quirin Schmidt, Kristina Nilsson Ekdahl, Bo Nilsson, Michael Fauler, Karl Josef Föhr, Markus Huber-Lang, David Alexander Christian Messerer

**Affiliations:** 1 Institute of Clinical and Experimental Trauma Immunology, University Hospital Ulm, 89081 Ulm, Germany; 2 Department of Hematology and Oncology, University Hospital Augsburg, 86156 Augsburg, Germany; 3 Institute of Applied Mathematics, Heidelberg University, 69120 Heidelberg, Germany; 4 Institute of Experimental and Clinical Pharmacology, Toxicology and Pharmacology of Natural Products, University of Ulm Medical Center, 89081 Ulm, Germany; 5 Rudbeck Laboratory, Department of Immunology, Genetics and Pathology, Uppsala University, 75185 Uppsala, Sweden; 6 Linnæus Center of Biomaterials Chemistry, Linnæus University, 39182 Kalmar, Sweden; 7 Institute of General Physiology, Ulm University, 89081 Ulm, Germany; 8 Department of Anesthesiology and Intensive Care Medicine, University Hospital Ulm, 89081 Ulm, Germany; 9 Department of Transfusion Medicine and Hemostaseology, Friedrich-Alexander University Erlangen-Nuremberg, University Hospital Erlangen, 91052 Erlangen, Germany

**Keywords:** anaphylatoxins, innate immunity, complement system, neutrophils, inflammation

## Abstract

The cellular and fluid phase-innate immune responses of many diseases predominantly involve activated neutrophil granulocytes and complement factors. However, a comparative systematic analysis of the early impact of key soluble complement cleavage products, including anaphylatoxins, on neutrophil granulocyte function is lacking. Neutrophil activity was monitored by flow cytometry regarding cellular (electro-)physiology, cellular activity, and changes in the surface expression of activation markers. The study revealed no major effects induced by C3a or C4a on neutrophil functions. By contrast, exposure to C5a or C5a des-Arg stimulated neutrophil activity as reflected in changes in membrane potential, intracellular pH, glucose uptake, and cellular size. Similarly, C5a and C5a des-Arg but no other monitored complement cleavage product enhanced phagocytosis and reactive oxygen species generation. C5a and C5a des-Arg also altered the neutrophil surface expression of several complement receptors and neutrophil activation markers, including C5aR1, CD62L, CD10, and CD11b, among others. In addition, a detailed characterization of the C5a-induced effects was performed with a time resolution of seconds. The multiparametric response of neutrophils was further analyzed by a principal component analysis, revealing CD11b, CD10, and CD16 to be key surrogates of the C5a-induced effects. Overall, we provide a comprehensive insight into the very early interactions of neutrophil granulocytes with activated complement split products and the resulting neutrophil activity. The results provide a basis for a better and, importantly, time-resolved and multiparametric understanding of neutrophil-related (patho-)physiologies.

## 1. Introduction

Neutrophil granulocytes (neutrophils) and the complement system serve as the vanguard of cellular and humoral innate immunity [1,2,3,4]. The complement cascade consists of over 50 proteins, mainly synthesized in the liver, and includes several zymogens [3,5]. Upon activation, most complement proteins are divided into specific cleavage products, which in turn orchestrate different effector functions. The three major effector functions are anaphylatoxin release, opsonization, and the formation of a membrane attack complex, resulting in inflammation, clearance of molecular danger, and cellular lysis [4,5,6]. Neutrophils are the most abundant cells of the innate immunity in the blood circulation [2,7]. Their activity can be promoted by numerous substances, including complement cleavage products such as complement factor 5a (C5a), interleukins such as interleukin 8 (IL-8), pathogen-associated molecular patterns (PAMPs) such as N-formylmethionyl-leucyl-phenylalanine (fMLF), lipid-derived mediators such as platelet-activating factor (PAF), and many others [2,7,8,9,10,11,12]. Upon activation, neutrophils respond with a defined change in cellular physiology and by exerting their main effector functions. The activity and the crosstalk of neutrophils and the complement system is a vital immunological process. On the one hand, neutrophil activity is crucially involved in clearing pathogens, cellular debris, and infected or degenerated cells. On the other hand, excessive and dysregulated complement and/or neutrophil activity can promote organ injury, and, ultimately, death [1,13,14,15,16,17,18].

The promotion of a proinflammatory response is traditionally ascribed to the anaphylatoxins C3a, C4a, and C5a [6,19,20]. However, for C3a, and in particular, C4a, it remains unclear whether and, if so, to what extent these factors are able to stimulate neutrophils [20,21,22]. By contrast, for C5a, there are numerous well-described effects on neutrophils: For example, C5a induces the depolarization of the membrane potential (MP) and increase in the intracellular pH (pH_i_) and glucose uptake (GlcU) [8,9,10]. Moreover, C5a alters the surface expression of several neutrophil activation markers, including complement receptors, for example, by inducing the upregulation of CD11b (part of the complement receptor 3 (CR3)) or by the downregulation of C5aR1 [23,24]. Moreover, C5a triggers neutrophil effector functions, such as chemotaxis, phagocytosis, the generation of radical oxygen species (ROS), and the formation of neutrophil extracellular traps (NETosis) [6,17]. Of note, C3a and C5a are converted in vivo by carboxypeptidases to desarginated forms (C3a des-Arg and C5a des-Arg, respectively) [25]. Previous data demonstrated that C5a des-Arg but not C3a des-Arg is capable of activating neutrophils, although the activation level by C5a des-Arg is comparatively mild in dependence of the used experimental context and the selected readouts [26,27].

The involvement of the complement–neutrophil axis has been demonstrated in several pathophysiologic sequelae in diseases, including rheumatoid arthritis, severe injuries, and sepsis [2,14,18,28,29]. For example, C5a can interact with the phagocytotic activity of neutrophils in patients with critically illness involving the inhibition of RhoA and the subsequent prevention of actin polymerization [17]. The therapeutic regulation of complement activity is an emerging concept for multiple diseases. Most interventions aim to target C3, C5, or their respective cleavage products, such as C5a. The inhibition of activated complement has already demonstrated notable success in clinical practice, for example, C3 and/or C5 inhibitors for paroxysmal nocturnal hemoglobinuria (PNH) [30] or targeting C1s in cold agglutination disease [31]. Moreover, complement-based interventions are discussed and evaluated in extended pathophysiologic aspects, including targeting C3 in oral inflammatory conditions [32] or in COVID-19-related acute inflammation [33,34]. Nonetheless, there remain many pathophysiological entities featuring the complement–neutrophil interaction without adequate interventional options [35]. Therefore, there is a need in translational research and clinical practice to better understand differential complement–neutrophil interactions, thereby allowing an improved definition of therapeutic targets as well as their subsequent biomarker-based monitoring.

Consequently, this study investigates the impact of key cleavage products, including classical anaphylatoxins C3a, C4a, C5a, and C5a des-Arg on neutrophils. Neutrophil activation is quantified in depth by analyzing cell physiology, activation markers, cellular effector functions, and complement receptors and regulators. Furthermore, the complement-related biomarkers of neutrophil activation are identified. Finally, the kinetics of complement-mediated activation is investigated using innovative near-real-time methods providing, for the first time, highly resolved multiparametric insights into neutrophil activation after stimulation with complement activation products.

## 2. Materials and Methods

### 2.1. Blood Sampling

Following ethical approval by the Local Independent Ethics Committee of Ulm University (#459/18), informed written consent was obtained from healthy female and male human volunteers with aged 21–38 without acute medication or recent change in chronic medication and without clinical signs of infection. Peripheral venipuncture was performed as described by the guideline of the World Health Organization [36]. Blood was collected into syringes containing 3.2% trisodium citrate or 16 IE/mL lithium–heparin (Sarstedt, Nümbrecht, Germany). All blood samples were maintained at room temperature and processed within 10 min.

### 2.2. Measurement of Membrane Potential, Intracellular pH, Glucose Uptake, and Cellular Shape

Purified leukocytes were used to determine MP, pH_i_, GlcU, and cellular shape. To this end, 15 mL whole blood was diluted with 15 mL 0.9% NaCl and gently layered upon 7.5 mL 5% dextran solution for sedimentation (30 min, room temperature) followed by hypotonic lysis (10 s of resuspension in distilled and sterile water), and the leukocytes were adjusted to 2 × 10^6^ cells/mL and stored in Hank’s Balanced Salt Solution with calcium and magnesium (HBSS^+/^^+^, Thermo Fisher Scientific, Darmstadt, Germany). Exemplary results of the purification process are shown in Supplementary Figure S1. All described buffers were adjusted to pH 7.3.

MP, pH_i_, and forward scatter area (FSC) were measured as described previously [9,11,12]. Leukocytes were stained with 50 nM bis(1,3-dibutylbarbituric acid) trimethine oxonol (DiBAC_4_(3), #D8189, Merck, Darmstadt, Germany, for MP), and 1 µM SNARF 5-(and-6)-carboxy-SNARF-1 (SNARF, #C1272, Invitrogen Thermo Fisher, Dreieich, Germany, for pH_i_) in HBSS and maintained in a light-protected water bath at 37 °C. After 20 min, cells were centrifuged (5 min, 340× *g*, room temperature) and resuspended in Roswell Park Memorial Institute-1640 with L-Glutamine and HEPES (RPMI^+/+^, Thermo Fisher Scientific) followed by another incubation period of 10 min with 50 nM DiBAC_4_(3) before stimulation and measuring. To measure GlcU, leukocytes were centrifuged after the above-described purification and resuspended in RPMI together with 30 µM 2-deoxy-2-([7-nitro-2,1,3-benzoxadiazol-4-yl]amino)-D-glucose (2NBDG, #72987, Sigma Aldrich, Steinheim, Germany) for 10 min in a light-protected water bath at 37 °C.

**Figure 1:**
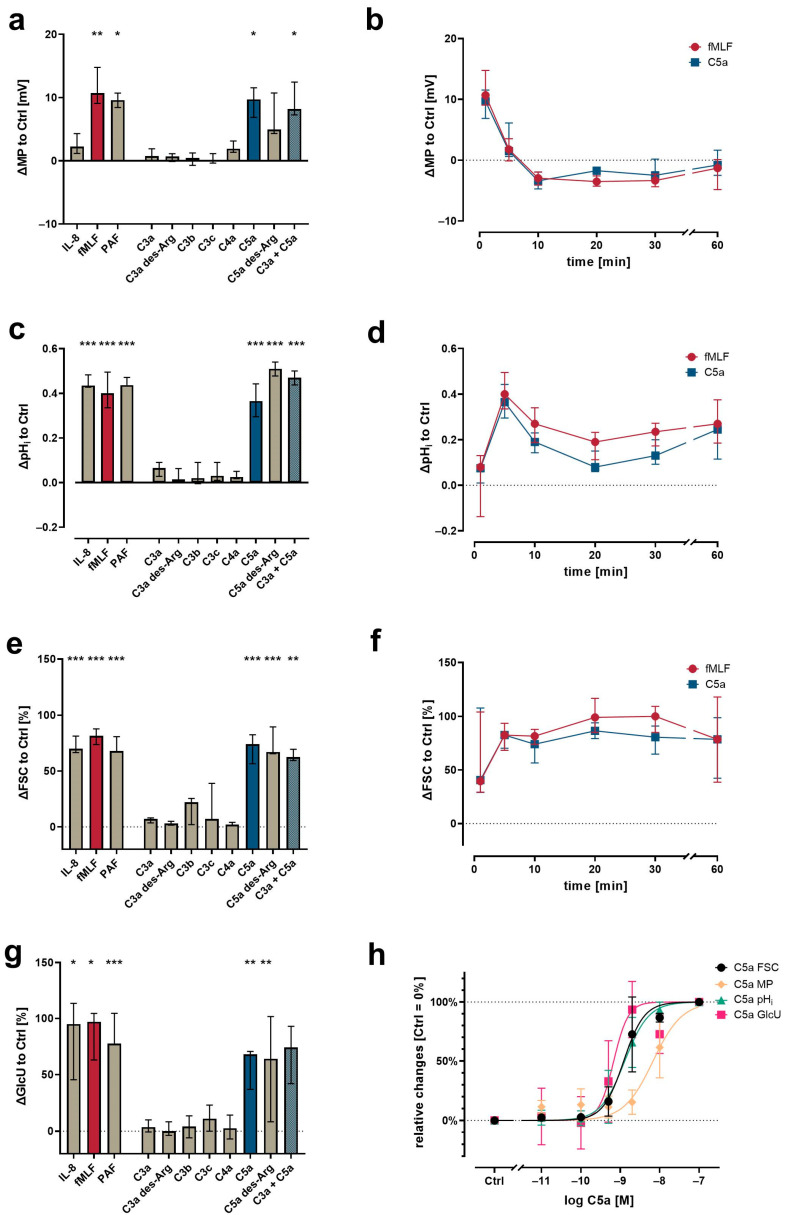
Neutrophil physiological response upon exposure to complement cleavage products with respect to changes (**a**) of membrane potential (MP, measured by change in DiBAC_4_(3) fluorescence) 1 min after stimulation, (**c**) intracellular pH (pH_i_, measured by change in SNARF fluorescence) 5 min after stimulation, (**e**) cellular shape as indicated by FSC 10 min after stimulation, and (**g**) glucose uptake (GlcU, measured by change in 2NBDG fluorescence) as measured after 10 min of stimulation. Time curves summarize for the change in (**b**) MP, (**d**) pH_i_, and (**f**) cellular shape. (**h**) Summary of the concentration-dependency of the C5a-induced effects. Results are normalized to unstimulated neutrophils (= 0% or 0). The following concentrations were used: 10 nM C5a, 10 nM C5a des-Arg, 10 µM fMLF, 50 ng/mL IL-8, 100 nM C3a, 100 nM C3a des-Arg, 100 nM C3b, 100 nM C3c, or 100 nM C4a. *n* = 6–8, median ± interquartile range. *, **, and *** = *p* < 0.05, < 0.01, and < 0.001, respectively. Kruskal–Wallis test with uncorrected Dunn’s test.

Following the indicated fluorescent dyes (either SNARF and DiBAC_4_(3) or 2NBDG), leukocytes were stimulated with 10 nM C5a (#A144, Complement Technology, Tyler, TX, USA), 10 nM C5a des-Arg (#A145, Complement Technology), 10 µM fMLF (#F3506, Sigma Aldrich), 50 ng/mL IL-8 (#I1645, Sigma Aldrich), or with 100 nM of the following complement cleavage products (all from Complement Technology): C3a (#A118), C3a des-Arg (#A119), C3b (#A114), C3c (#A116), or C4a (#A106). In the screening process, the concentrations as commonly used by us and others were chosen to exceed approximately 10-fold the concentrations reported for cellular activation, and to achieve plasma concentrations in the upper range as reported during systemic inflammation of the respective cleavage products [8,9,10,11,19,37,38,39,40]. After 1, 5, 10, 20, 30, and 60 min, leukocytes were analyzed by flow cytometry using a BD Canto II (BD Biosciences, Heidelberg, Germany). Following the exclusion of doublets by checking the linearity of FSC-A vs. FSC-H (normally <2%), polymorphonuclear granulocytes (PMN) mainly consisting of neutrophils were identified by FSC-A and side scatter area (SSC-A) (Supplementary Figure S1). For each measurement shown in Figure 1, a minimum of 5000 neutrophils were analyzed with a high flow rate (120 µL/min). The analysis of MP and pH_i_, including the corresponding mathematical corrections and calibration curves, were performed exactly as described in [9].

### 2.3. Measurement of Neutrophil Cellular Function and Surface Marker Expression

**Figure 2:**
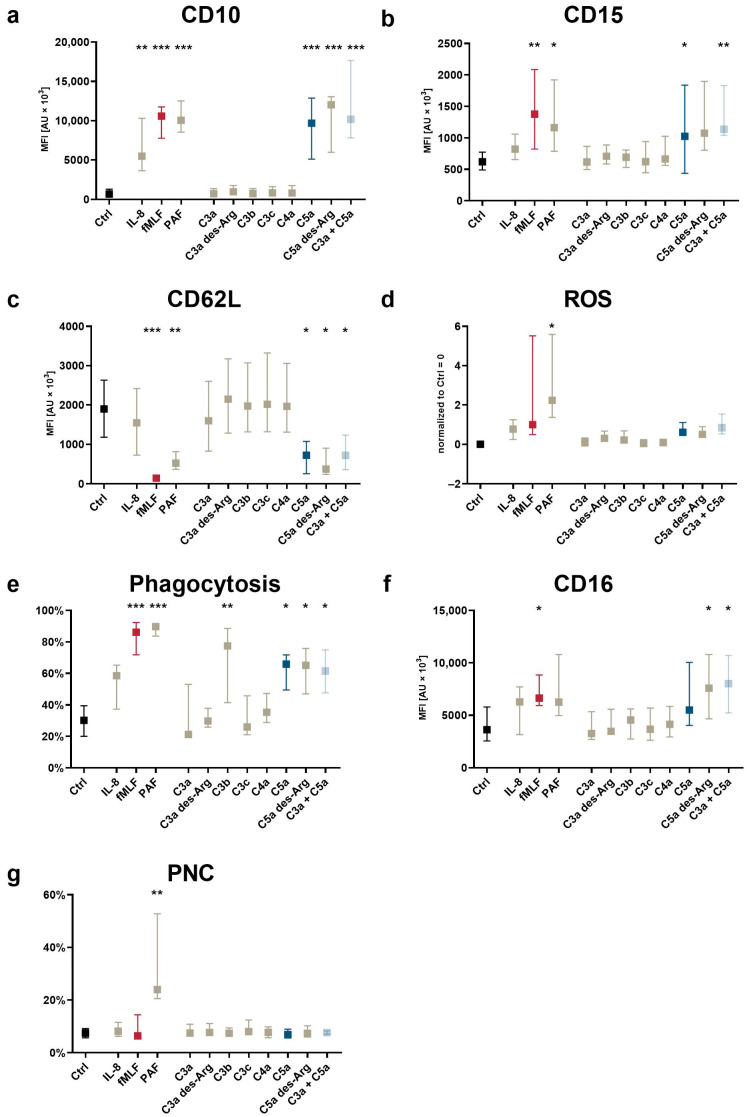
Changes in neutrophil activation markers and cellular functions after exposure to complement cleavage products with respect to (**a**) CD10, (**b**) CD15, (**c**) CD62L, (**d**) the generation of ROS, (**e**) phagocytotic activity, (**f**) CD16, and (**g**) the formation of platelet–neutrophil complexes (PNCs). Y-axis reports the median fluorescence intensity (MFI) for all CD molecules, the percent positive for PNC formation and phagocytosis, and the increase in ROS production normalized to unstimulated neutrophils (= 0). The following concentrations were used: 10 nM C5a, 10 nM C5a des-Arg, 10 µM fMLF, 50 ng/mL IL-8, 100 nM C3a, 100 nM C3a des-Arg, 100 nM C3b, 100 nM C3c, or 100 nM C4a. n = 6–8, median ± interquartile range. *, **, and *** = *p* < 0.05, < 0.01, and < 0.001, respectively. Kruskal–Wallis test with uncorrected Dunn’s test.

**Figure 3:**
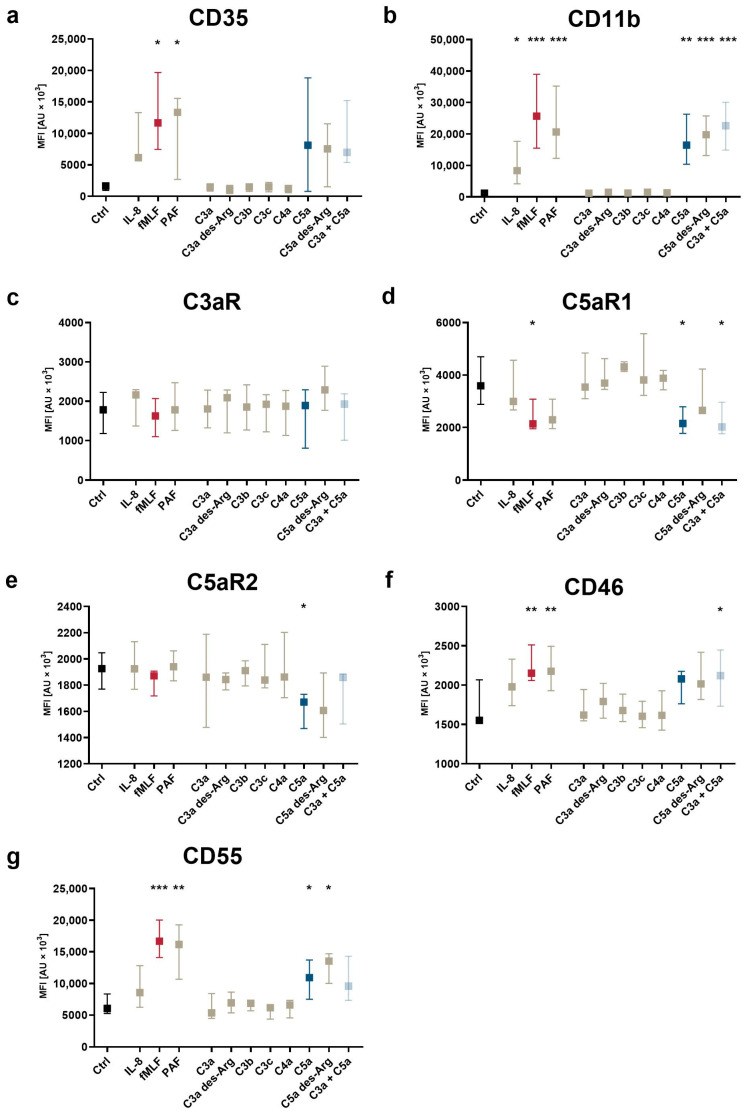
Changes after exposure to complement cleavage products of the neutrophil complement-related receptors and regulators with respect to (**a**) CD35, (**b**) CD11b, (**c**) C3aR, (**d**) C5aR1, (**e**) C5aR2, (**f**) CD46, and (**g**) CD55. Y-axis reports median fluorescence intensity (MFI). The following concentrations were used: 10 nM C5a, 10 nM C5a des-Arg, 10 µM fMLF, 50 ng/mL IL-8, 100 nM. *, **, and *** = *p* < 0.05, < 0.01, and < 0.001, respectively. Kruskal–Wallis test with uncorrected Dunn’s test.

**Figure 4:**
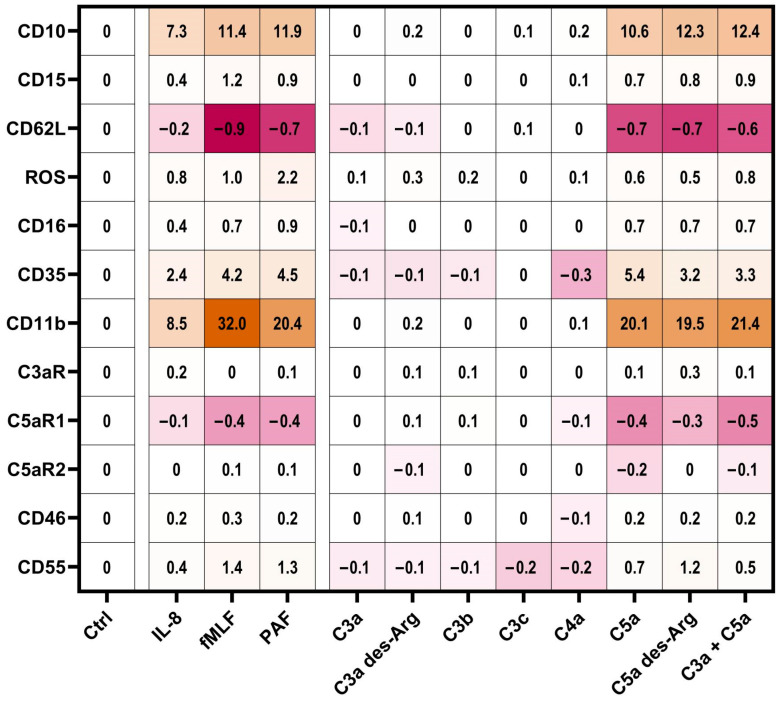
Summary of the neutrophil response. Results report the median change of stimulated cells normalized to the respective ctrl (= 0). Cellular response in the form of an upregulation is indicated by different shades of orange and downregulation is indicated by different shades of pink. The following concentrations were used: 10 nM C5a, 10 nM C5a des-Arg, 10 µM fMLF, 50 ng/mL IL-8, 100 nM C3a, 100 nM C3a des-Arg, 100 nM C3b, 100 nM C3c, or 100 nM C4a. *n* = 6–8.

**Figure 5:**
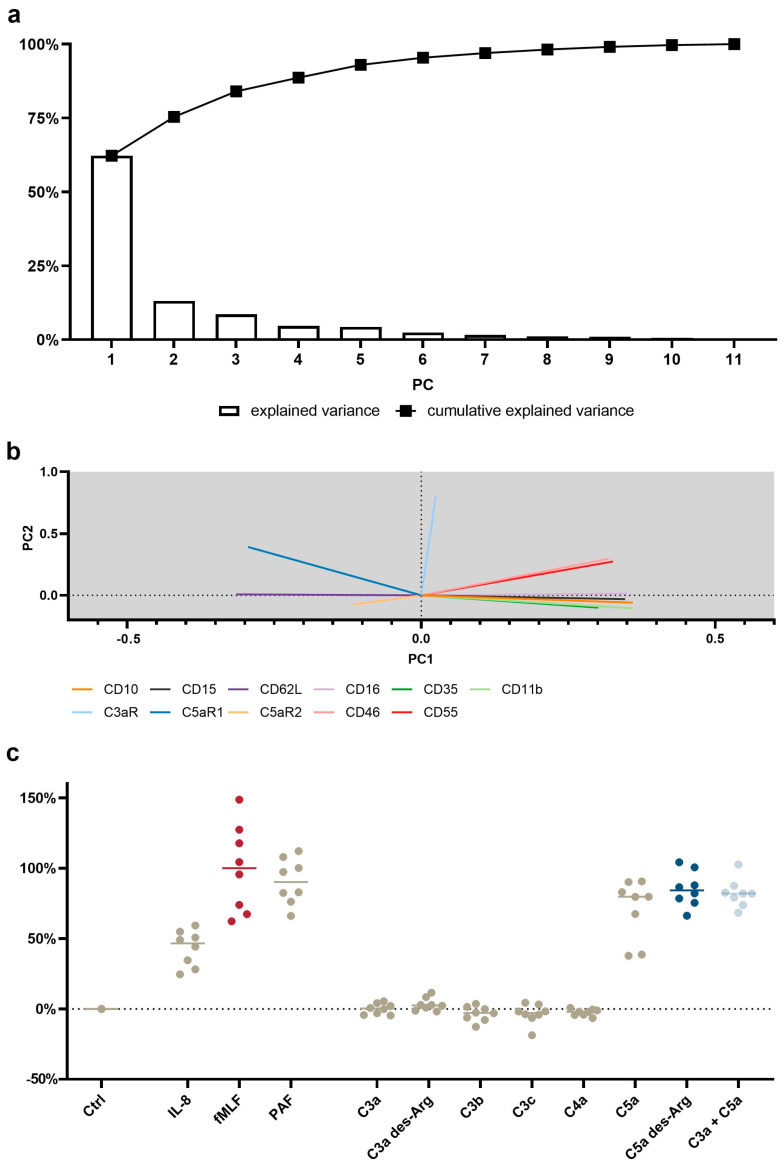
Principal component analysis (PCA) of the neutrophil response after stimulation with C3a, C4a, or C5a or other inflammation-related stimuli. (**a**) Scree plot reporting the explained variance per principal component (PC) and the cumulative explained variance. (**b**) Loading plot reporting the vector of the respective parameter of PC1 and PC2. The scaling of the X-axis and Y-axis represent the explained variance of 62% and 13%, respectively. (**c**) First PC normalized to 0% = unstimulated neutrophils and 100% = median of fMLF-stimulated neutrophils. The following concentrations were used: 10 nM C5a, 10 nM C5a des-Arg, 10 µM fMLF, 50 ng/mL IL-8, 100 nM C3a, 100 nM C3a des-Arg, 100 nM C3b, 100 nM C3c, or 100 nM C4a. *n* = 8, median with scatter plot.

For neutrophil activation marker measurement, 10 µL citrate-anticoagulated whole blood were diluted with phosphate-buffered saline with calcium and magnesium (PBS^+/+^, Thermo Fisher Scientific) to a total volume of 50 µL and stimulated with either fMLF, IL-8, PAF (500 ng/mL, #18779, Cayman Chemical, Ann Arbor, MI, USA), C5a, or the different complement cleavage products using concentrations as indicated, while PBS^+/+^ served as the buffer control. Cell suspensions were incubated with fluorescent-labelled reagents and antibodies for 15 min in a light-protected water bath at 37 °C. Neutrophil cellular functions, such as ROS, phagocytotic activity, and platelet–neutrophil complexes (PNCs), were measured using 10 µL heparin anti-coagulated whole blood and incubated for 30 min at 37 °C. To this end, the subsequently listed antibodies and their respective isotype controls were used (all from BioLegend, San Diego, CA, USA, except CD55 from BD Biosciences, Franklin Lakes, NJ, USA): CD62L (L-selectin, PE, final conc.: 62.5 ng/mL, #304806; isotype: #400112), CD10 (neprilysin, PE-Cy7, 120 ng/mL, #312214; isotype: #400118), CD11b (CR3, APC, 7.5 ng/mL, #101212; isotype #400612), CD16 (FcγRIII, PerCP, 200 ng/mL, #302030; isotype: #400148), CD15 (SSEA-1, FITC, 50 ng/mL, #301904; isotype: #401606), CD88 (C5aR1, APC, 134.4 ng/mL, #344310; isotype: #400222), C5L2 (C5aR2, PE, 10.6 µg/mL, #342404; isotype: #400212), C3aR (PE-Cy7, 66 ng/mL, #345808; isotype: #400326), CD35 (CR1, FITC, 1 µg/mL, #332406; isotype: #400108), CD46 (membrane cofactor protein, FITC, 2 µg/mL, #315304; isotype: #400108), and CD55 (DAF, BV510, 1 µg/mL, #563027; isotype: #742678). PNC formation was determined using CD41 (BV605, 100 ng/mL, #303742; isotype: #400612) and analyzed as described previously [12]. ROS production was measured using CellROX Deep Red reagent (5 µM, #C10422, Thermo Fisher Scientific) and the ability for phagocytosis was determined by adding fluorescent labelled phagocytosis beads (#18339-10, Polysciences Inc., Warrington, PA, USA). Subsequently, the complete content was transferred into 5 mL polystyrene round-bottom tubes (#352052, Corning Science México S.A. de C.V., Reynosa, Mexico) containing 1 mL 1:10 diluted FACS Lysing Solution (#349202, BD Biosciences) and incubated for 30 min at room temperature in the dark. Samples were centrifuged at 340× *g* for 5 min, the supernatant was discarded, and the cells were finally resuspended in PBS without calcium or magnesium (PBS^−/−^, Thermo Fisher Scientific) containing 1% bovine serum albumin (BSA, #A8022, Sigma-Aldrich). Samples were stored at 4 °C in the dark until measurement within one hour with a medium flow rate (60 µL/min). For each measurement shown in Figure 2, Figure 3, Figure 4 and Figure 5, a minimum of 3000 neutrophils were analyzed using a BD Lyric flow cytometer (BD Biosciences).

### 2.4. Near-Real-Time Measurement of C5a-Induced Response Kinetics of Neutrophils

In total, 20 µL citrate-anticoagulated whole blood was diluted with 30 µL PBS^−/−^ containing the following antibodies (all from BioLegend): CD11b (final concentration: 8 µg/mL), CD35 (8 µg/mL), CD10 (8 µg/mL), CD16 (16 µg/mL), CD62L (1 µg/mL), CD45 (Pacific Blue, 4 µg/mL, #368540), and CD66b (APC-Cy7, 8 µg/mL, #305126). Cells were stained in a light-protected water bath at 37 °C for 10 min. The stained blood was transferred into a 5 mL polystyrene round-bottom tube with a total volume of 1650 µL PBS^+/+^. Prior to the transfer, the tube was perforated at a height of 4.5 cm using a soldering station (BASETech, Hirschau, Germany) forming a small whole with a diameter of approximately 7–10 mm. Following a resting period of 2 min in the water bath at 37 °C, the sample was placed in the manual acquisition port of the BD Lyric flow cytometer. The tube was surrounded by a temperature-controlled heating unit (#TC-124A Handheld Temperature Controller, 64–1545, Warner Instruments, Holliston, MA, USA) set to 37.3 °C. Acquisition with a medium speed was started and continued for 1320 s. After 55 s, a peripheral venous catheter (Vasofix^®^ Safety 1.30 × 45 mm G 18, #4268130S-01, B.Braun, Melsungen, Germany) was placed in the polystyrene tube during the acquisition. Attached to the peripheral venous catheter was a 1 mL syringe (BD Plastipak^TM^, #303172, Becton Dickinson S.A. Madrid, Spain) containing 150 µL stimulation mixture. Following a 60 s baseline measurement, the content of the syringe was rapidly injected into the polystyrene tube followed by a bolus of 200–300 µL air to ensure the proper mixing of the stimulants. The stimulation mixture consisted of C5a in PBS^+/+^, which, after injection, resulted in a final C5a concentration of 10 nM.

### 2.5. Determination of the Half-Maximal Effective Concentrations

Half-maximal effective concentration (EC_50_) was determined by fitting a Hill equation to normalized response values at various C5a concentrations (range of 10 pM–100 nM). For this purpose, the C5a-induced response was standardized by subtracting fluorescence signals of resting cells stimulated with PBS^+/+^ and normalizing to signals from cells stimulated with 100 nM C5a. The EC_50_ was determined from a nonlinear regression fit applying a variable slope model (y = Bottom + (Top − Bottom)/(1 + 10^((LogEC_50_ − x) × HillSlope)) x = log of concentration, y = response) with min/max constraints (bottom = 0, top = 1). Data analysis and model fitting were performed with GraphPad Prism 8 (GraphPad Software Inc., San Diego, CA, USA).

### 2.6. Principal Component Analysis (PCA) for Dimensionality Reduction in Data from Multicolor Flow Cytometry

A training set was generated using stimulus versus control ratios of the median fluorescence intensities of neutrophils applying the formula: (stimulated/control) – 1. All stimuli (PBS^+/+^ as control, fMLF, IL-8, PAF, C3a, C3a des-Arg, C3b, C3c, C4a, C5a, C5a des-Arg, and C3a + C5a) applied to samples from eight donors were included using the subsequent listed parameters: CD11b, CD35, CD10, CD16, CD62L, CD15, CD46, CD55, C3aR, C5aR1, and C5aR2. On the basis of the resulting dataset, the z-score of each entry within the respective parameter was calculated to normalize the input data. Subsequently, the principal components (PCs) were calculated by single value decomposition of the normalized input data (which comprised the whole training data set). On the basis of the PCs, the output values were calculated using a linear combination of the PCs and the training set. The output values were normalized, with unstimulated neutrophils set to 0% and fMLF-stimulated neutrophils to 100%. The resulting loading matrix is reported in Supplementary Table S2 and the first two PCA loading vectors can be visualized in Figure 1b.

### 2.7. Analysis of Kinetics of Neutrophil Activation

To analyze the near-real-time kinetics of whole blood without further fixation by multicolor flow cytometry according to the staining protocol as described above, a stepwise gating approach was conducted. First, a CD45-threshhold was established to exclude contaminating erythrocytes. Second, doublets were excluded based on the linearity of FSC-A and FSC-H. Third, neutrophils were identified as SSC^high^, CD16^high^, CD45^high^, and CD66b^high^ cells. On the basis of this gating strategy, a median of 23.2 (21.5|27.1) neutrophils per seconds was measured.

The identified neutrophils were further analyzed using a custom written, python-based flow cytometry analytics software “BFlow” (BFlow Project, www.bflow.science, last accessed 2 September 2022). For this purpose, a moving median analyzing nine consecutive neutrophils was calculated for each parameter. The C5a-induced change in neutrophil phenotype within the first 15 min was characterized regarding the time elapsed to the half-maximal response (Figure 2a), time to maximal slope (Figure 2b), the maximal slope relative to the baseline (Figure 2c), and the ratio of the plateau to the baseline (Figure 2d).

For the analysis of the resulting kinetics, the response of the neutrophils was divided into three parts (seconds in brackets refer to the total acquisition time with neutrophils being stimulated after 60 s): baseline (10–50 s), initial stimulation (60–90 s), and response kinetic (90–960 s). The baseline was defined as the median of the measurement within the seconds 10–50 s. The response kinetic was fit as a polynomial of the fifth order. The polynomial was fitted using the moving median of seconds 65–1140. However, because it is difficult to distinguish effects induced by either C5a or mixing of the cells, directly after stimulation, the seconds 60–90 (first 30 s after stimulation) were approximated by a linear curve starting from the baseline and ending at the polynomial-derived value of 90 s. Examples of the used fits are summarized in Supplementary Figure S5.

### 2.8. Statistical Analysis and Presentation

Data were analyzed using GraphPad Prism 8 and Microsoft Excel (version 16.32, Microsoft Corporation, Redmond, WA, USA). Data are reported as the median with whiskers indicating interquartile range, if not indicated otherwise. In the manuscript, data are reported as median (25th quantile|75th quantile). The present study followed a two stepped statistical approach: First, the listed stimuli were screened in blood from at least seven independent donors. Outliers were identified based on lab records (potential technical errors, such as low cell number in tube) and/or discussion of at least two authors (A.E.P.S. and D.A.C.M.). Disagreements were resolved by consulting a third author (L.W.). In the PCA, eliminated values were substituted by the mean of the particular parameter. The generated screening data were analyzed comparing the median fluorescence intensity (MFI) or the amount of % positive cells as appropriate with the respective control sample using Kruskal–Wallis test with uncorrected Dunn’s test. This approach ensures conservative testing regarding missing potential positive stimuli, but also may include false-positive results due to repeated testing. To address this issue, a confirmatory dataset for the resulting hit regarding C5a was generated when generating the respective concentration–response curve. Here, the two groups of the results of 10 nM C5a and respective control cells were compared using the Mann–Whitney test to corroborate the findings of the screening (Supplementary Table S3).

## 3. Results

### 3.1. Differential Changes in Neutrophil Cell Physiology upon Stimulation with Various Complement Cleavage Products

In general, the activation of neutrophils is linked to changes in MP, pH_i_, cellular shape, and GlcU, as shown previously [8,9,10,11,12,41]. Using neutrophils within a population of purified leukocytes, the screening of various complement activation products revealed a unique response profile for C5a and C5a des-Arg, whereas the C3 cleavage products (C3a, C3b, C3c, and C3a des-Arg) and C4a had no detectable effect at a concentration of 100 nM (Figure 3). At 10 nM, C5a and C5a des-Arg elicited a depolarization of the neutrophil MP comparable 10 µM fMLF (Figure 3a). The addition of 100 nM C3a to 10 nM C5a did not further enhance (or decrease) cellular depolarization compared to C5a alone. Similarly, C5a and C5a des-Arg increased the pH_i_, the cellular size as measured by FSC, and the glucose uptake (Figure 3c,e,g). Time courses of C5a-induced changes in cellular physiology followed a clear time pattern, which was similar to the response after stimulation with fMLF (Figure 3b,d,f). The comparisons of the magnitude of the complement-mediated cellular response is summarized in Figure 3a,c,e at the time points of the peaks for fMLF stimulation. The response of neutrophils to all measured complement cleavage products were analyzed after 1, 5, 10, 30, and 60 min. However, no further relevant response was captured during this period (data not shown). Neutrophil activation by C5a regarding MP, pH_i_, FSC, and GlcU was concentration-dependent (Figure 3h). Of note, a higher EC_50_ concentration of C5a was necessary to induce depolarization (6.8 nM, 95% CI 4.7–9.8 nM), when compared (*p* = 0.008, Mann–Whitney test, analyzing samples individually shown as summarized in Figure 3h) to the EC_50_ determined for alkalization (1.3 nM, 95% CI 1.0–1.6 nM).

### 3.2. The Impact of Complement Cleavage Products on Neutrophil Phenotype as Well as Altered Cellular Effector Functions

Neutrophil activation is defined by altered cellular effector functions and reflected by changes in the surface expression of activation markers and complement receptors as well as altered cellular effector functions [23,42]. The analysis of activation markers and cellular effector functions revealed a marked response of neutrophils in whole blood to C5a and C5a des-Arg for indicators associated with chemotactic activity, including CD10 upregulation (Figure 4a), CD15 upregulation (Figure 4b), and CD62L downregulation (Figure 4c). None of the screened complement cleavage products enhanced PNC formation (Figure 4g). C5a-induced increased ROS production, although at a lower level as by either fMLF or PAF (Figure 4d). C5a and C5a des-Arg induced increased the expression of the phagocytosis receptor CD16 and an enhanced phagocytotic activity (Figure 4f,e). As expected, the latter was also increased after exposure to C3b, known as the classical opsonin. All C5a-induced changes were unaffected when co-stimulating neutrophils with C3a and C5a.

### 3.3. Modulation of Complement Receptors and Regulators on Neutrophils by C5-Cleavage Products

**Figure 6:**
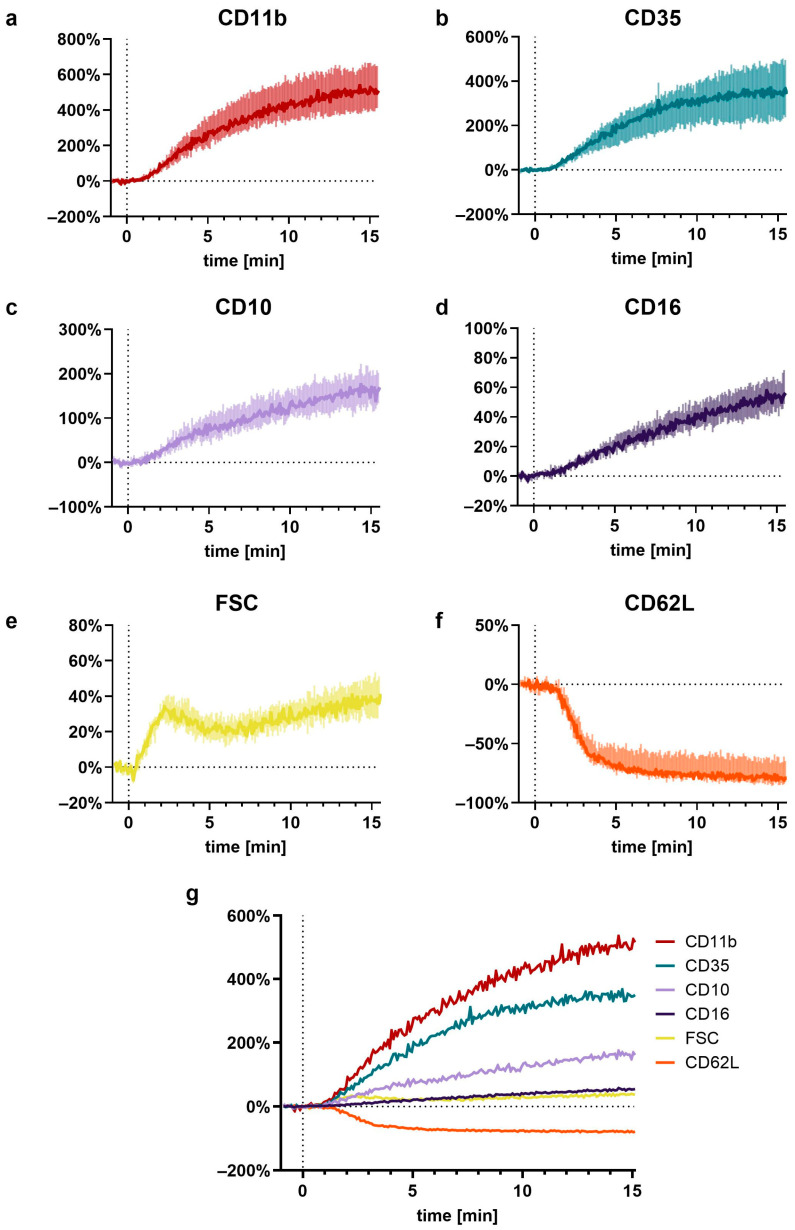
Near-realtime description of the C5a-induced (10 nM) effects on neutrophils for (**a**) CD11b, (**b**) CD35, (**c**) CD10, (**d**) CD16, (**e**) FSC, (**f**) CD62L, and (**g**) a summary of all these factors. Reported are the changes normalized to neutrophils prior to stimulation (= 0%) as the median with shaded areas indicating the interquartile range, *n* = 7–8.

Subsequently, the impact of complement cleavage products on the surface expression of complement receptors and regulators on neutrophils in whole blood was analyzed. Both C5a and C5a des-Arg increased CD11b, CD35, CD46, and CD55 expression, while both decreased the expression of C5aR1 expression (Figure 5). Of note, C5a-induced alterations of the MFI did not greatly affect the percentage of positive neutrophils within the respective marker as summarized in Supplementary Table S1. For example, neutrophil stimulation with C5a reduced CD62L surface expression, but the majority of neutrophils remained positive for this marker as indicated by respective isotype control staining. C3aR expression remained unaffected after stimulation with either complement cleavage products or fMLF, IL-8, or PAF (Figure 5c). Figure 3, Figure 4 and Figure 5 report the statistical results of the screening procedure. As indicated in the methods section, the findings regarding C5a were confirmed using a second dataset reported in Supplementary Table S3. Figure 6 summarizes the relative change in neutrophil cell physiology, activation markers, effector functions, complement receptors, and regulators after stimulation with complement cleavage products or respective positive controls. Of note, the simultaneous stimulation of neutrophils by C3a and C5a neither increased nor decreased neutrophil activation beyond the level of stimulation with C5a alone.

### 3.4. PCA Reveals a Common Neutrophil Activation Pattern

To identify a general signature of neutrophil activation, a panel of eleven antibodies was applied to neutrophils stimulated by eleven activators. Given the complexity of the generated data, dimensionality reduction was performed by means of PCA. The first three PCs were able to identify 84% of the variance in neutrophil activation. The first PC explained 62% of the variance, with changes in CD11b, CD10, and CD16 being identified as the most important markers of neutrophil activity (Figure 1a, Supplementary Table S2). Figure 1b visualizes the first two principal component vectors of the loading matrix, summarizing how each characteristic parameter influenced the respective principal component. The loading summarizes surface antigens, which responded similarly upon stimulation with complement cleavage products. Smaller angles between vectors indicate the similar behavior of the respective surface marker. 

Orthogonal angles indicate no dependence of the respective antigens. The longer the vector in the *x*-axis and/or *y*-axis, the stronger is its influence in PC1 and/or PC2, respectively. 

On the basis of the PCA, a neutrophil stimulation index was generated ranging from 0% (unstimulated neutrophils) to 100% (fMLF-stimulated neutrophils). Here, the median activation by C5a and C5a des-Arg was 80% (46%|88%) and 84% (76%|97%), respectively (Figure 1c). The full results for PC1–5 are reported in detail in Supplementary Figure S4.

### 3.5. In-Depth Analysis of the C5a-Induced Response

Finally, a detailed investigation of the C5a-induced cellular activation pattern was conducted analyzing the concentration-dependency and the kinetics in a near-real-time manner. All C5a-induced effects displayed a concentration–response relationship. 

For example, the EC_50_ for CD11b upregulation was calculated to be 6.7 nM (95% confidence interval (CI): 5.1–8.6 nM) and for CD10 upregulation 5.5 nM (95% CI: 4.2–7.1 nM) (Supplementary Figures S2 and S3).

**Figure 7:**
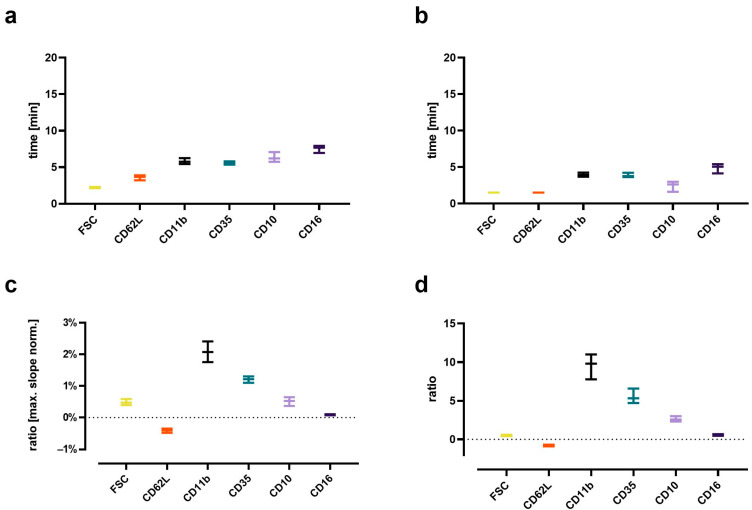
Analysis of the kinetics of the C5a-induced (10 nM) changes in neutrophils. (**a**) Time to when half of the maximal change in the respective value occurred, (**b**) time at the maximum slope, (**c**) maximal slope normalized to the respective baseline, and (**d**) the ratio of the change defined as the plateau 15 min after stimulation normalized to the baseline = 0. *n* = 7–8, median ± interquartile range.

The C5a-induced response of neutrophils demonstrated a well-defined time pattern (Figure 7). All measured responses (CD11b, CD35, CD10, CD16, FSC, and CD62L) started within minutes after stimulation with C5a. However, while CD35, CD11b, CD10, and CD16 changed gradually, reaching a plateau after 10–15 min, the response of FSC and CD62L were much more rapid and attained a plateau within the first 5 min. Comparing the C5a-induced response in regard to the time (for example, until reaching its maximal slope, time to complete half of the maximal change, maximal slope, and its overall ratio), it revealed highly uniform responses in neutrophils, irrespective of the donors investigated (Figure 2). Among the analyzed surface markers, CD11b displayed the most rapid and strongest response in neutrophils after exposure to C5a.

## 4. Discussion

In the present study, C5a and C5a des-Arg elicited a rapid, multivariate response in neutrophils in contrast to C3a, C3a des-Arg, C3b, C3c, and C4a, which did not induce such effects. The predominant role of C5a and C5a des-Arg is in accordance with previous findings [8,10,24,41,42]. The response of neutrophils induced by C5a and C5a des-Arg was comparable to effects elicited by fMLF or PAF single readouts as well as their combination in principal components. However, neither C5a nor C5a des-Arg promoted PNC formation, which implicated that a platelet–neutrophil interaction is not a hallmark of complement activity and, therefore, is likely to be neither a diagnostic marker nor largely involved in complement-mediated diseases, at least not in the whole blood assay used here. Interestingly, a study using isolated neutrophils, platelets, and serum found that complement activation appears to be involved in PNC formation on endothelial cells [43].

The C5a-induced response of neutrophils was further assessed and confirmed to be concentration-dependent with EC_50_s ranging over approximately 10^–8^–10^–9^ M. This finding is accordance with other studies reporting an EC_50_ for C5a-binding to C5aR1 of approximately 1 nM and to C5aR2 of approximately 3–10 nM [19,37,38]. Previous studies reported C5a concentrations of approximately 10 nM during inflammation [19,39,44,45]. Interestingly, a higher EC_50_ concentration of C5a was determined to induce depolarization when compared to alkalization. A current widely accepted hypothesis is that neutrophil depolarization is associated with the activity of the NADPH oxidase (NOX), which is crucially involved in ROS generation [13,46,47,48,49]. NOX activity results in electron extrusion, which must be compensated, which is likely mediated by proton extrusion via voltage-gated proton channels and/or sodium-proton exchanger 1 (NHE1) activity [13,46,49]. However, surprisingly, C5a-induced alkalization occurred at lower concentrations than the depolarization. Currently, we cannot exclude that this is due to limitations of the measurement methods, for example, the response time of the used fluorescent probes to monitor depolarization and alkalization. Further studies with direct methods, for example, combining patch-clamp with the measurement of extracellular proton concentrations, are warranted to elucidate this issue.

Concomitant with a previous study [9], this study analyzes in-depth the rapid kinetic of the C5a-induced response. Interestingly, the C5a-induced response of neutrophils appears to have three response types: For depolarization, there is an initial peak within the first 2 min, with a return to baseline after 5 min. Alkalization, changes in FSC, and CD62L shedding can be observed starting within the first minutes, reaching plateaus after approximately 5 min. By contrast, the C5a-induced CD11b, CD35, CD10, and CD16 upregulation occurred more gradually and was delayed. In this context, further studies need to clarify whether these dynamics are similar using lower C5a concentrations and to link the different kinetics observed in cellular physiology and phenotype to specific functional consequences. 

This study has several strengths and limitations. The role of C5a is highlighted synchronically by analyzing the response of neutrophils regarding cellular biological parameters and changes in cellular phenotype. Although the findings were generated solely by flow cytometry, this is a well-established and clinically applied method to analyze neutrophils. In addition, our findings are in accordance with previous studies, for example, also reporting C5a but not C3a-related effects on neutrophils [21,50]. Moreover, flow cytometry allows the non-invasive analysis of non-adhering neutrophils. Most readouts were captured using diluted whole blood, which allowed the stimulation of the neutrophils after venipuncture. This decision was based avoiding purification steps that might artificially activate neutrophils and to take the short lifespan of neutrophils into account. Moreover, this ensured (to a certain degree) the presence of plasma proteins potentially interacting with complement cleavage products, thereby more closely mimicking the physiological situation. For MP, pH_i_, FSC, and GlcU, the isolation of leukocytes was chosen because the removal of erythrocytes by lysing agents, for example, ammonium chloride, might interact with cellular parameter, in particular the pH_i_. The further purification of neutrophils within the leukocyte population was omitted to reduce potential artificial activation and delay due to the isolation process. Of note, GlcU was determined indirectly using the fluorescent dye 2NBDG, the validity of which as a surrogate for GlcU was recently challenged [51]. Further studies need to confirm some specific findings by other methods, for example, depolarization by the patch-clamp technique, cellular activation by analyzing humoral activation markers of neutrophils, or alterations of cell metabolism by metabolic flux analysis. Furthermore, we analyzed blood from young healthy volunteers, thereby potentially overlooking the effects of certain pre-existing complement activation during acute and/or chronic comorbidities. Moreover, our analysis focused on central complement cleavage products; however, we neither analyzed all complement proteins nor reflected the fact that complement system activation results in a parallel increase in the concentration of several complement cleavage products, nor were complement components bound to any surfaces (which would provide clustering of C3 opsonins). In addition, this study used an innovative approach to analyze the response of neutrophils in near-real-time. Finally, the conducted PCA revealed a specific change in the neutrophil phenotype upon activation. The corresponding loading values of the used activation markers and their resulting vectors on the loading plot contribute to a better understanding of hallmarks of neutrophil activity and to identify potential relevant and redundant markers for the immunomonitoring of complement-driven neutrophil activation. 

## 5. Conclusions

C5a and C5a des-Arg but neither C3a nor C4a induced a multiparametric response in neutrophils as demonstrated for cell physiological parameters, neutrophil phenotype, and cellular effector functions. The findings including the reported EC_50_ values of C5a and the C5a-induced response kinetic of neutrophils have implications for complement-driven pathologies, for example, regarding the design and evaluation of an intervention. Further studies need to assess the findings in other cell types, such as other leukocytes and platelets.

## Data Availability

All data will be made available upon reasonable request by emailing the corresponding author.

## Supplementary Materials

The following supporting information can be downloaded at: https://www.mdpi.com/article/10.3390/cells11203297/s1, Supplemental Table S1. Summary of the neutrophils positive for the used surface markers; Supplemental Table S2. Principal component analysis; Supplemental Table S3. Statistical analysis for C5a-induced effect on neutrophils; Supplemental Figure S1. Representative result of the leukocyte isolation procedure and summary of the applied gating strategy; Supplemental Figure S2. Concentration-response curves; Supplemental Figure S3. Calculated EC_50_ for the activation of neutrophils elicited by C5a; Supplemental Figure S4. Result of the principal component analysis; Supplemental Figure S5. Representative curve fitting for neutrophil kinetics after stimulation with C5a.
