# Supplementary material for: Modulation of Neutrophil Activity by Soluble Complement Cleavage Products—An In-Depth Analysis
Source: Cells. 2022 Oct 20;11(20):3297. doi: 10.3390/cells11203297 (PMC9600402; doi:10.3390/cells11203297)
Supplement: Supplementary file 1 [file cells-11-03297-s001.zip › cells-1943928-supplementary.pdf]

<table>
<tr><th></th><th>Ctrl</th><th>fMLF</th><th>PAF</th><th>C3c</th><th>C3a</th><th>C3a des-Arg</th><th>C5a</th><th>C5a des-Arg</th><th>C4a</th><th>C3b</th><th>IL-8</th><th>C3a + C5a</th></tr>
<tr><td>CD10</td><td>59.3 (51.3 | 84.4)</td><td>99.0 (98.5 | 99.3)</td><td>99.4 (99.0 | 99.6)</td><td>65.3 (53.3 | 85.8)</td><td>70.7 (59.7 | 84.1)</td><td>75.2 (74.9 | 89.1)</td><td>99.0 (97.3 | 99.2)</td><td>98.9 (97.1 | 99.3)</td><td>72.4 (67.1 | 90.5)</td><td>66.3 (55.5 | 84.5)</td><td>94.6 (88.5 | 98.5)</td><td>99.2 (98.6 | 99.5)</td></tr>
<tr><td>CD15</td><td>99.4 (98.7 | 99.8)</td><td>99.5 (95.7 | 99.8)</td><td>99.6 (94.9 | 99.7)</td><td>99.0 (88.9 | 99.7)</td><td>99.6 (97.3 | 99.6)</td><td>99.1 (92.7 | 99.8)</td><td>99.2 (89.5 | 99.9)</td><td>99.2 (95.3 | 99.6)</td><td>98.6 (94.3 | 99.8)</td><td>99.3 (95.0 | 99.6)</td><td>99.3 (95.1 | 99.7)</td><td>99.6 (98.6 | 99.9)</td></tr>
<tr><td>CD62l</td><td>99.0 (91.7 | 99.7)</td><td>40.6 (29.1 | 47.7)</td><td>90.1 (77.3 | 93.2)</td><td>98.8 (95.9 | 99.6)</td><td>99.3 (96.7 | 99.5)</td><td>99.1 (98.0 | 99.5)</td><td>92.7 (73.2 | 98.4)</td><td>86.3 (72.9 | 97.7)</td><td>99.2 (96.7 | 99.4)</td><td>98.9 (97.7 | 99.5)</td><td>98.1 (93.4 | 99.4)</td><td>93.4 (85.3 | 98.1)</td></tr>
<tr><td>CD16</td><td>99.3 (98.5 | 99.5)</td><td>99.6 (99.2 | 99.8)</td><td>99.5 (99.4 | 99.6)</td><td>99.3 (98.9 | 99.4)</td><td>99.4 (98.1 | 99.6)</td><td>99.4 (98.6 | 99.5)</td><td>99.4 (99.0 | 99.7)</td><td>99.3 (98.6 | 99.6)</td><td>99.4 (98.7 | 99.6)</td><td>99.5 (99.2 | 99.6)</td><td>99.4 (98.8 | 99.6)</td><td>99.6 (99.3 | 99.7)</td></tr>
<tr><td>CD35</td><td>84.5 (76.2 | 87.1)</td><td>99.1 (93.4 | 99.8)</td><td>99.1 (89.2 | 99.9)</td><td>80.4 (53.8 | 90.3)</td><td>75.9 (57.5 | 87.6)</td><td>64.7 (39.8 | 88.9)</td><td>99.2 (92.6 | 99.6)</td><td>89.8 (80.7 | 99.2)</td><td>76.2 (60.1 | 88.1)</td><td>83.3 (51.4 | 87.5)</td><td>88.8 (85.2 | 99.5)</td><td>94.8 (89.2 | 99.9)</td></tr>
<tr><td>CD11b</td><td>97.7 (94.3 | 99.1)</td><td>99.9 (99.8 | 100.0)</td><td>99.9 (99.8 | 100.0)</td><td>98.6 (94.6 | 99.3)</td><td>97.3 (94.9 | 98.1)</td><td>98.8 (96.8 | 99.0)</td><td>99.9 (99.8 | 99.9)</td><td>99.9 (99.7 | 100.0)</td><td>98.9 (95.9 | 99.2)</td><td>98.1 (94.8 | 99.1)</td><td>99.7 (97.5 | 100.0)</td><td>99.9 (99.9 | 100.0)</td></tr>
<tr><td>C3aR</td><td>84.4 (77.9 | 92.4)</td><td>87.2 (80.2 | 94.1)</td><td>88.4 (76.2 | 93.9)</td><td>89.1 (83.1 | 95.0)</td><td>90.5 (82.3 | 96.3)</td><td>94.7 (85.2 | 98.0)</td><td>92.4 (66.3 | 96.2)</td><td>97.0 (88.9 | 98.5)</td><td>83.9 (73.1 | 96.7)</td><td>93.6 (86.5 | 98.0)</td><td>94.4 (88.9 | 96.8)</td><td>93.8 (82.7 | 97.5)</td></tr>
<tr><td>C5aR1</td><td>99.1 (90.0 | 99.9)</td><td>98.6 (96.8 | 100.0)</td><td>99.6 (95.6 | 99.9)</td><td>99.8 (99.3 | 100.0)</td><td>99.7 (97.7 | 100.0)</td><td>99.8 (99.7 | 99.9)</td><td>99.1 (97.9 | 99.8)</td><td>99.9 (99.4 | 99.9)</td><td>99.8 (97.8 | 99.9)</td><td>99.8 (99.3 | 99.9)</td><td>99.8 (99.7 | 99.9)</td><td>99.7 (99.5 | 99.8)</td></tr>
<tr><td>C5aR2</td><td>99.7 (92.7 | 100.0)</td><td>99.3 (98.2 | 99.9)</td><td>99.8 (99.6 | 99.9)</td><td>99.8 (99.7 | 100.0)</td><td>99.8 (99.2 | 100.0)</td><td>100.0 (99.8 | 100.0)</td><td>99.9 (99.4 | 100.0)</td><td>99.9 (99.6 | 100.0)</td><td>100.0 (99.5 | 100.0)</td><td>100.0 (99.6 | 100.0)</td><td>99.9 (99.7 | 100.0)</td><td>99.7 (99.4 | 100.0)</td></tr>
</table>

**CD46**	99.9 (90.6 ǀ 100.0)	99.2 (97.6 ǀ 100.0)	99.9 (99.6 ǀ 100.0)	99.9 (99.7 ǀ 100.0)	100.0 (99.8 ǀ 100.0)	100.0 (100.0 ǀ 100.0)	100.0 (99.9 ǀ 100.0)	100.0 (99.8 ǀ 100.0)	100.0 (99.9 ǀ 100.0)	100.0 (99.8 ǀ 100.0)	100.0 (99.9 ǀ 100.0)	100.0 (99.9 ǀ 100.0)
**CD55**	94.6 (90.1 ǀ 98.6)	98.5 (97.9 ǀ 99.9)	99.1 (98.7 ǀ 99.4)	96.6 (94.3 ǀ 98.7)	94.2 (92.1 ǀ 99.3)	97.4 (95.5 ǀ 98.9)	99.0 (96.1 ǀ 99.5)	99.3 (97.9 ǀ 99.5)	96.1 (92.9 ǀ 99.1)	98.5 (94.4 ǀ 99.5)	98.1 (95.8 ǀ 99.5)	99.1 (97.4 ǀ 99.4)

Supplementary Table S1. Summary of the neutrophils positive for the used surface markers in relationship to a respective isotype. n = 6 – 8, median (first quartile ǀ third quartile).

PC	CD10	CD15	CD16	CD62L	CD35	CD11b	C3aR	C5aR1	C5aR2	CD46	CD55	explained variance [%]	explained variance cumulative [%]
1	0.36	0.35	0.35	−0.31	0.30	0.36	0.02	−0.29	−0.12	0.32	0.33	62.29	62.29
2	−0.06	−0.03	0.01	0.01	−0.10	−0.11	0.81	0.39	−0.08	0.30	0.27	13.09	75.38
3	0.03	0.06	−0.02	−0.08	−0.09	0.07	−0.03	0.07	0.97	0.15	0.10	8.64	84.03
4	0.09	0.24	0.34	0.65	0.41	0.19	0.10	0.28	0.10	−0.25	−0.17	4.67	88.70
5	0.10	0.39	0.31	0.16	−0.72	0.00	0.14	−0.25	−0.03	0.04	−0.32	4.32	93.02
6	−0.05	−0.21	0.01	0.53	0.14	−0.35	−0.03	−0.49	0.04	0.52	0.11	2.40	95.43
7	0.62	−0.45	0.01	−0.15	0.12	−0.02	0.25	−0.06	0.07	0.04	−0.54	1.58	97.01
8	0.14	−0.01	0.30	−0.04	−0.10	−0.24	−0.48	0.59	−0.11	0.47	−0.08	1.16	98.17
9	−0.24	0.32	−0.49	0.00	0.16	0.34	0.03	0.06	−0.05	0.46	−0.49	0.92	99.09
10	0.51	−0.06	−0.47	0.37	−0.31	0.34	−0.15	0.13	−0.10	0.02	0.34	0.61	99.71
11	0.34	0.55	−0.34	−0.07	0.17	−0.63	0.03	0.01	0.02	−0.14	0.02	0.29	100.00

Supplementary Table S2. Principal component analysis (PCA) as well as its related first five principal components (PC) and loading matrix analyzing the change of indicated markers in stimulated neutrophils as described in the methods section.

	p-value screening	p-value confirmation	Ctrl	C5a
**CD10**	0.0003	0.0079	569.6 (549.3 ǀ 725.6)	4524 (3976 ǀ 5164)
**CD15**	0.1953	0.0159	788.2 (582 ǀ 872.2)	1313 (982.5 ǀ 1486)
**CD62L**	0.0134	0.5476	2583 (1645 ǀ 3420)	2014 (1488 ǀ 2724)
**ROS**	0.2759	0.9999	959.6 (693.7 ǀ 2021)	1102 (660.3 ǀ 1853)
**Phagocytosis**	0.0229	0.1255	18.89 (16.26 ǀ 23.20)	24.64 (20.61 ǀ 41.72)
**CD16**	0.2112	0.0411	4360 (2985 ǀ 6877)	8415 (4813 ǀ 10177)
**CD35**	0.1451	0.0649	1727 (1450 ǀ 2268)	12368 (5577 ǀ 18881)
**CD11b**	0.0018	0.0079	915.4 (738.9 ǀ 1310)	7807 (7255 ǀ 12919)
**C5aR1**	0.0275	0.2086	3782 (3696 ǀ 5094)	2937 (2630 ǀ 4566)
**C5aR2**	0.0439	0.0043	1829 (1729 ǀ 2013)	1604 (1485 ǀ 1656)
**CD46**	0.0657	0.0175	1548 (1526 ǀ 1646)	1844 (1758 ǀ 1934)
**CD55**	0.0382	0.0087	5236 (4512 ǀ 6834)	8124 (6877 ǀ 11556)
**FSC**	0.0006	0.0079	79420 (75756 ǀ 83090)	127612 (123216 ǀ 145483)
**MP**	0.0165	0.0262	4437 (3980 ǀ 5727)	6365 (6054 ǀ 8046)
**pH_i**	0.0001	0.0079	7.50 (7.43 ǀ 7.58)	7.91 (7.89 ǀ 7.97)
**GlcU**	0.0039	0.0079	3668 (3191 ǀ 4072)	6088 (5581 ǀ 7191)

Supplementary Table S3. Comparison of resulting p-values of the screening process (corresponding results are reported in Figures 2 and 3) and the confirmation approach including the original data. MP is reported as $DiBAC_4(3)$ fluorescence, pH_i determined by SNARF fluorescence, and GlcU is quantified by 2NBDG fluorescence. The statistical approach is reported in detail in the method section using Kruskal-Wallis test with uncorrected Dunn's test for the screening process and Mann Whitney to confirm the findings. $n = 6 – 8$.

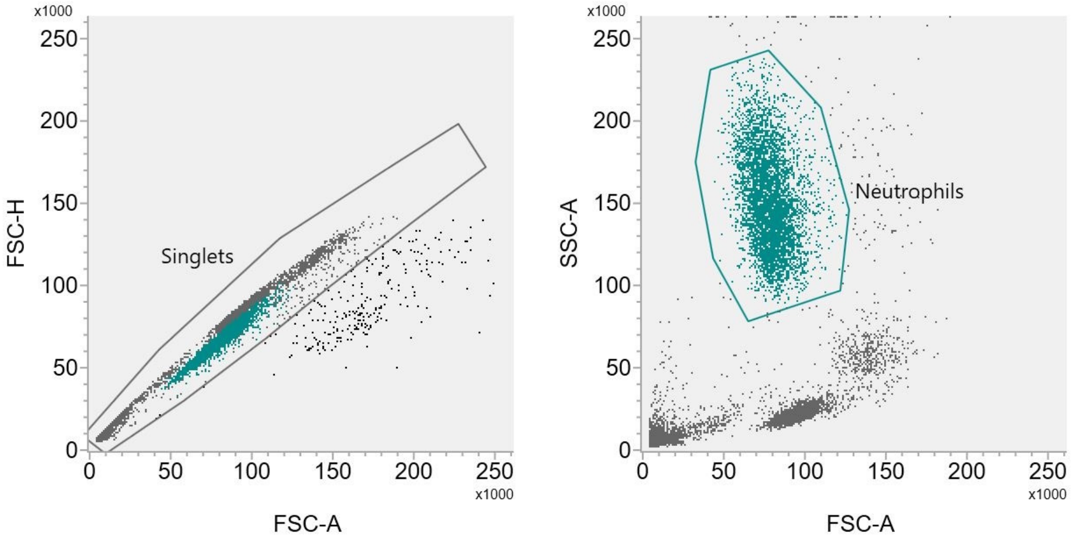

Supplementary Figure S1. Representative result of the leukocyte isolation procedure and summary of the applied gating strategy. Following identification of singlets (here: 98.1% of parent population) by checking linearity of FSC-A vs. FSC-H polymorphonuclear granulocytes (here referred to as neutrophils: 41.6% of parent population) mainly consisting of neutrophils were identified by FSC-A and SSC-A.

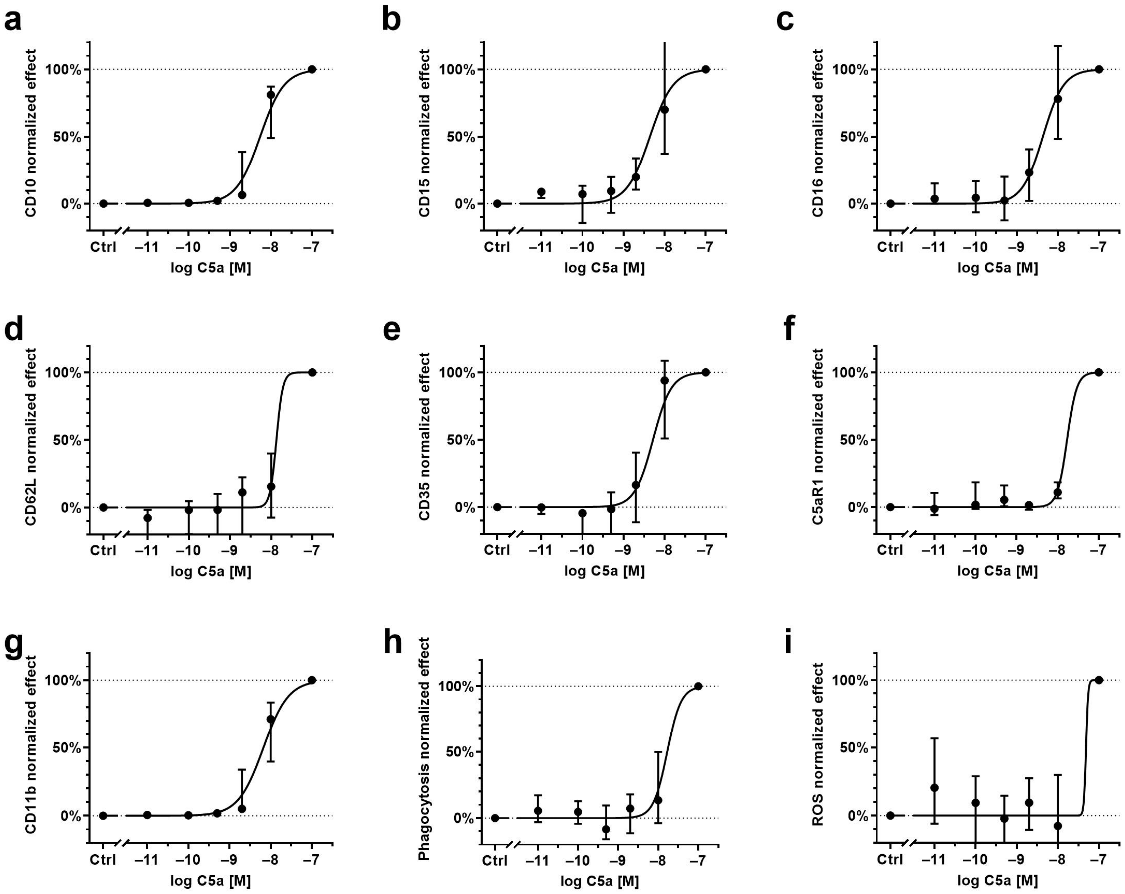

Supplementary Figure S2. Concentration-response curves of cellular function and neutrophil phenotype after stimulation with C5a. (**a**) CD10, (**b**) CD15, (**c**) CD16, (**d**) CD62L, (**e**) CD35, (**f**) C5aR1, (**g**) CD11b, (**h**) Phagocytosis and (**i**) ROS. Curves were normalized to PBS$^{+/+}$ as control. $n = 6 – 7$, median ± interquartile range.

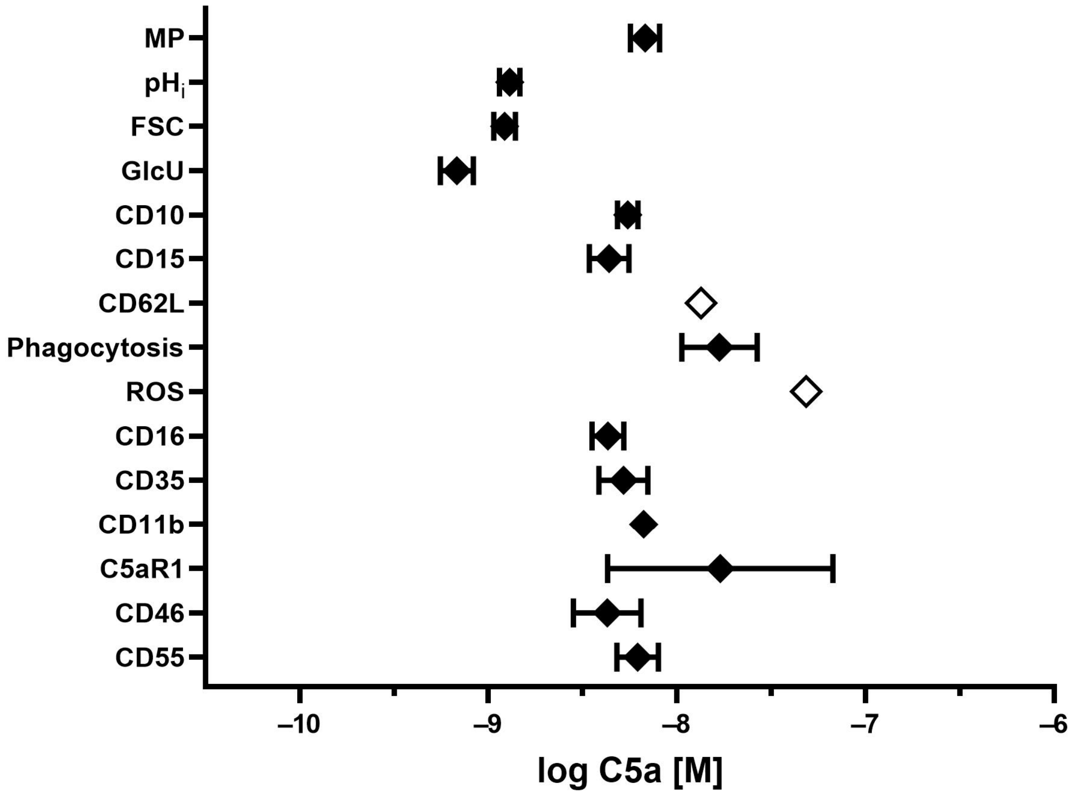

Supplementary Figure S3. Calculated EC_{50} for the activation of neutrophils elicited by C5a. $n = 6 - 7$, mean ± standard error of the mean of the EC_{50}.For empty diamonds, confidence intervals could not be calculated.

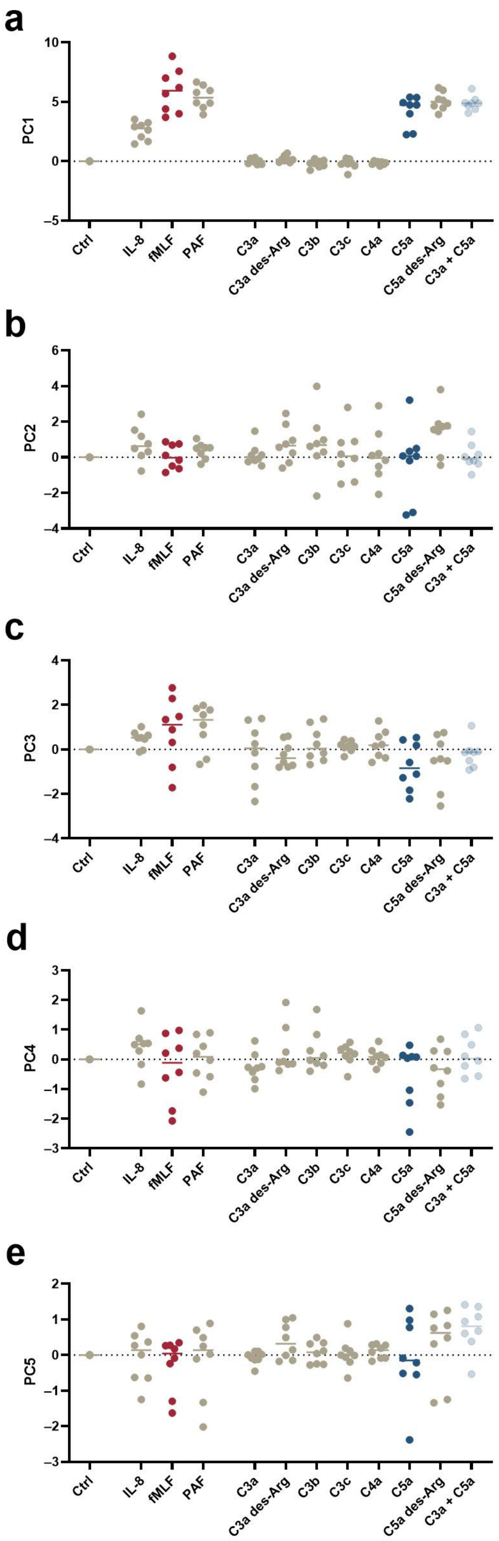

Supplementary Figure S4. Result of the principal component analysis (PCA) evaluating the first principal components (PC). The PCA was conducted by analyzing the change in neutrophil phenotype elicited by the respective stimulus as listed on the Y-axis. (**a**–**e**) Unstimulated neutrophils were set to 0. The corresponding loading matrix is reported in Supplementary Table S2. n = 8.

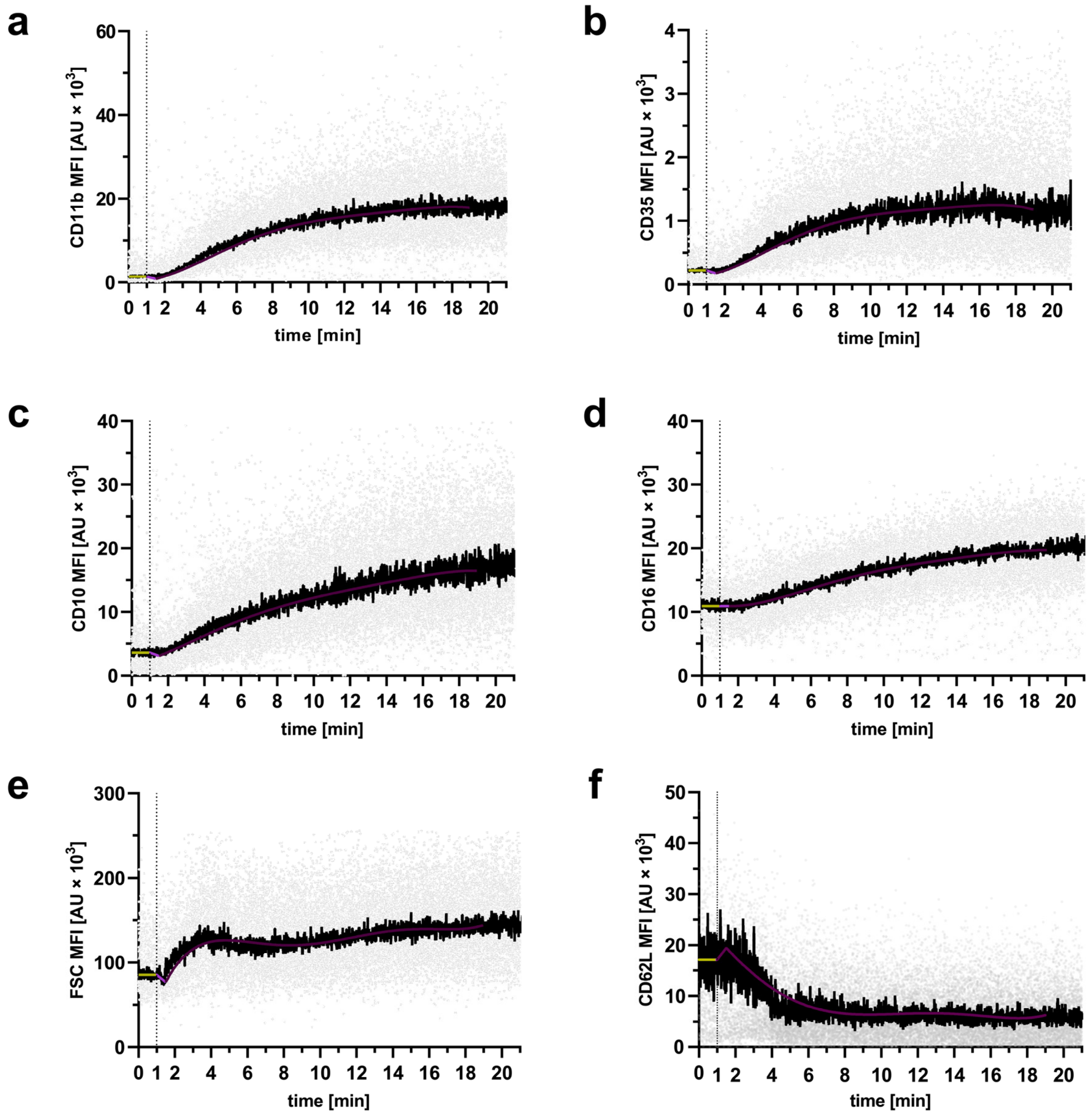

Supplementary Figure S5. Curve fitting for (**a**) CD11b, (**b**) CD35, (**c**) CD10, (**d**) CD16, (**e**) FSC, and (**f**) CD62L from a representative experiment. Gray dots indicate single cells. The black line shows a moving median analyzing nine consecutive neutrophils. The yellow line indicates the baseline, pink indicates the first 30 seconds as a linear approximation, and violet displays the used polynomial as described in the methods section.
